# Nest building in titmice Paridae: Selectivity in bryophyte use

**DOI:** 10.1002/ece3.9852

**Published:** 2023-03-08

**Authors:** Knut Rydgren, Bendik Indreeide, Tore Slagsvold, Helene M. Lampe

**Affiliations:** ^1^ Department of Environmental Sciences Western Norway University of Applied Sciences Sogndal Norway; ^2^ Tyristubbveien 3 Oslo Norway; ^3^ Department of Biosciences CEES, University of Oslo Oslo Norway

**Keywords:** behavioral ecology, bird nests, bryophytes, cavity‐nesting birds, nest material

## Abstract

In many bird species, reproductive success is dependent on nest quality. However, detailed data on nest composition are scarce, and quantitative analyses have generally used only rough categories, without species identification. Bryophytes dominate the nests of many passerine bird species, but little is known about whether birds have preferences for certain species. In this study, we determined the bryophyte species composition in nests of blue tits *Cyanistes caeruleus* and great tits *Parus major* in a forest near Oslo, Norway. We also sampled the abundance of the bryophyte species in plots on the forest floor surrounding a subset of the great tit nests. Blue tits and great tits both used 15 bryophyte species as nest materials, mainly the same pleurocarpous species but in different proportions. The tits preferred highly branched bryophyte species, i.e., *Pleurozium schreberi*, *Rhytidiadelphus squarrosus,* and *Sanionia uncinata* but avoided common forest floor bryophyte species that are sparsely branched*.* Great tits clearly collected bryophyte species selectively. We also found that bryophyte species content in great tit nests in the same nest box in different years was very similar. Our results also indicated that the great tits collected bryophyte nest materials close to their nests, mostly within 5 m, supporting the view that collecting nest materials is costly. We review several hypotheses to explain why the tits prefer certain species of bryophytes as nest materials. These include handling costs and their suitability as structural materials. We recommend field experiments to test specific hypotheses and to study whether preferences are heritable.

## Graphical abstract

Although many bird species use bryophytes abundantly for nest materials, we have little knowledge of which species they use, and even more whether birds have preferences for certain species. We show that both blue tits and great tits mostly use the same bryophyte species as nest material, and mainly highly branched bryophyte species. We also show that great tits clearly collected bryophyte species selectively and that they most likely collected bryophyte nest materials close to their nests, mostly within 5 m, supporting the view that collecting nest materials is costly.

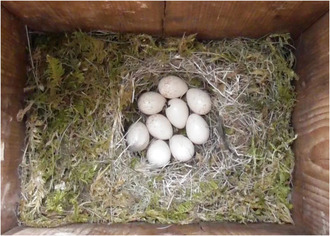

## 1 INTRODUCTION

Most birds build a nest for reproduction, and its basic function is to protect the eggs, chicks, and incubating and brooding parent (Hansell, 2000). Nests may provide an optimal microclimate, reducing heat loss, and protecting the contents from predators, ectoparasites, and pathogens (Clark & Mason, 1985; Mainwaring et al., 2014; Mennerat et al., 2009). However, nest building appears to be costly for the parents (Mainwaring & Hartley, 2013), due to the time and energy expended in flying and collecting materials and in constructing the nest itself (Bailey et al., 2016; Nudds & Bryant, 2000). Thus, there may be a trade‐off between nest quality and the costs of nest construction (Mainwaring et al., 2014; Mainwaring & Hartley, 2013). In many species, such as titmice Paridae, only the female builds the nest, and she usually puts on body mass during the nest‐building period to prepare for egg laying. This makes her particularly vulnerable to avian raptors (Slagsvold & Dale, 1996). Because of these costs, the nest and nest‐building activity may function as sexual signals of individual quality (extended phenotype), which in turn may affect parental investment (García‐Navas et al., 2015; Järvinen & Brommer, 2020) and hence avian evolution (Fang et al., 2018).

The general structure of bird nests comprises shape, size, composition, and lining (Perez et al., 2020). Different bird species use a variety of materials that may differ from one part of the nest to another (Hansell, 2000). Although nests of particular species are usually identifiable by humans (Aasen & Slagsvold, 2020; Dickinson et al., 2022; Hansell, 2000), nest design is a trait that varies considerably, particularly among but also within species (Biddle et al., 2018; Breen et al., 2016; Briggs & Deeming, 2021; Mainwaring et al., 2014). The most typical differences among species may be heritable (Aasen & Slagsvold, 2020) but studies of lining materials and nest depth in blue tits *Cyanistes caeruleus* have shown low heritability (Järvinen et al., 2017; O'Neill et al., 2018).

The plant materials used by birds have generally only been divided into rough categories, such as grass, ferns, lichens, and mosses (e.g., Biddle et al., 2018; Briggs et al., 2019; Britt & Deeming, 2011; Deeming & Mainwaring, 2015; Dickinson et al., 2022) rather than identified to species. The choice of nest materials may be important for reproduction and for the risk of nest and adult predation, and may thus have great evolutionary potential to respond to selective pressures (Perez et al., 2020). We should therefore utilize the extra information available by identifying the specific items used (e.g., Briggs & Deeming, 2021, Briggs & Deeming, 2016; Camacho‐Alpízar et al., 2021;Glądalski et al., 2021 ; Wesołowski & Wierzcholska, 2018).

The availability of nest materials in the immediate surroundings of a nest site may influence nest composition. Already in selecting a suitable nest site, the availability of nest material may be one of the important factors for the birds (Mainwaring et al., 2014). Prevailing evidence suggests that birds construct their nests opportunistically using nest material in proportion to availability (Briggs & Deeming, 2021; Lambrechts et al., 2017). However, because of the importance of the materials used, one would expect birds to be selective. Indeed, some evidence exists that birds select specific plant materials for their nests, in particular aromatic plants that may give protection against parasitic organisms (Petit et al., 2002), and that they use specific bryophytes for this purpose (Glądalski et al., 2021; Wesołowski & Wierzcholska, 2018).

Bryophytes (mosses and liverworts) are one of the main material types used in nests of passerine birds (Breil & Moyle, 1976; Briggs et al., 2019; Briggs & Deeming, 2021; Glime, 2017a). In the present study, we determined the bryophyte species composition in nests of two common cavity‐nesting birds, the blue tit and the great tit *Parus major*. Both species use large amounts of bryophytes in their nests, but usually only a few dominant species (Alambiaga et al., 2020; Britt & Deeming, 2011; Glądalski et al., 2016). The bryophytes used by the two tits may be fairly similar (Glądalski et al., 2021), or in other cases quite different (Wesołowski & Wierzcholska, 2018). In general, blue tits forage is higher above the ground than great tits (Slagsvold & Wiebe, 2007; Suhonen et al., 1994), which may affect where they collect bryophytes. Blue tits seem to have a stronger preference for epiphytic bryophytes, whereas great tits have a stronger preference for ground‐living or epigeic bryophytes (Glądalski et al., 2021; Henze, 1962; Wesołowski & Wierzcholska, 2018).

Two studies in Poland showed that both blue and great tits collected bryophytes selectively (Glądalski et al., 2021; Wesołowski & Wierzcholska, 2018). However, in both cases, only bryophytes growing within 10 m of the tit nest sites were included, and only species identity was recorded, not their relative abundance in the local area. We used a similar study design but improved it by quantifying the amounts of the various bryophytic species found both in a number of great tit nests and in plots on the ground doubling the distance to within 20 m of each great tit nest site.

We addressed four questions. First, we asked whether the species composition of blue tits and great tits nest differed, and if so whether blue tits collected more epiphytic bryophytes than great tits, while great tits collected more ground‐living (epigeic) species. The design also allowed a more detailed comparison of the amounts of each bryophyte species found in the great tit nests and their abundances in the surrounding sample plots. Second, we therefore tested whether bryophyte choice was random. Third, we compared the species composition of bryophytes in the same nest box (used by great tits) between different years, with the prediction that the content would be more similar between the same boxes than between different boxes, given that the bryophyte abundances within a local forest area remain relatively stable between years without disturbance (Rydgren et al., 2004). Finally, we analyzed the probable distances from the nest site at which great tits had collected bryophytes predicting that they would fly as short distances as possible to reduce time and energy building nest (Mainwaring & Hartley, 2013).

## 2 MATERIALS AND METHODS

### 2.1 Study area and study species

The study area of ca. 72 ha (altitude 150–200 m) consists of mixed coniferous and deciduous forest in Sørkedalen valley (59°59′ N, 10°38′ E) near Oslo, Norway. It is situated in the southern boreal zone and the slightly suboceanic section (Moen, 1999). Mean annual precipitation for the normal period 1991–2020 at Blindern 7 km further SE is 837 mm, with the peak in autumn, and mean temperatures for the same normal period are 6.2°C in April, 11.4°C in May and 15.3°C in June (https://seklima.met.no/).

**FIGURE 1:**
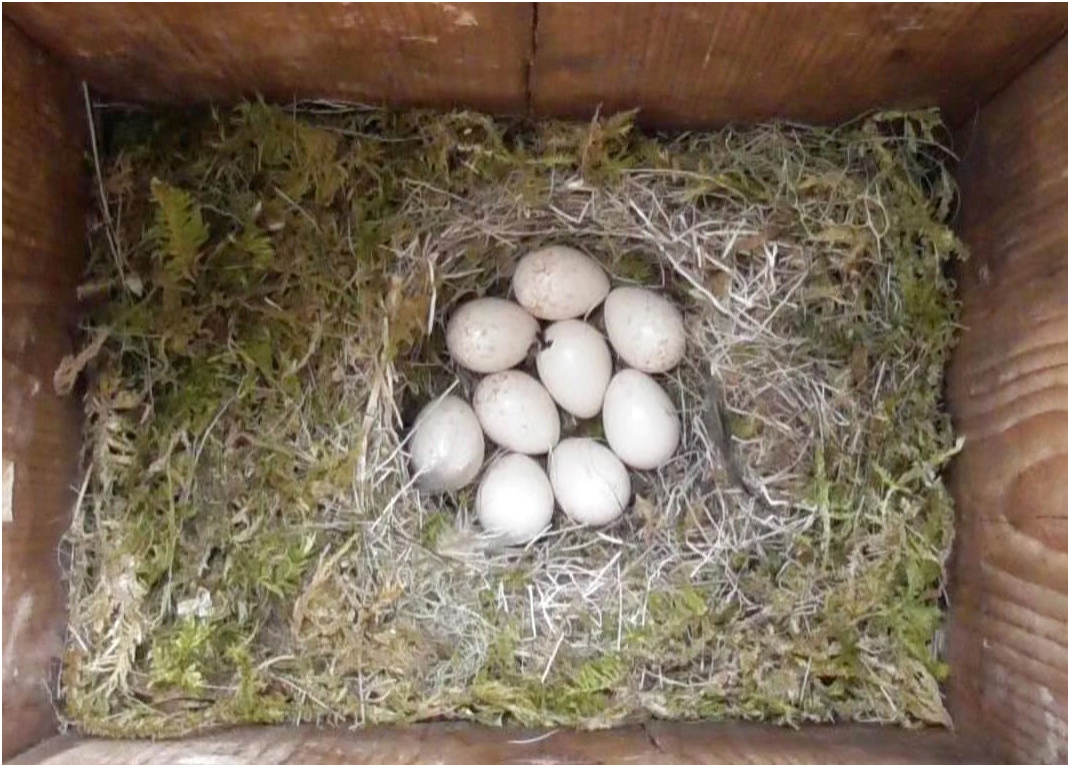
Blue tit nest with nine eggs photographed in the incubation period. The nest has a base layer of moss with a nest cup lined with hairs. Photo credits: Tore Slagsvold.

The study area contained approximately 300 nest boxes with an entrance diameter of 32 mm, attached to tree trunks about 1.5 m above the ground and about 50 m apart. The tits most commonly using the nest boxes were blue tits (Figure 1) and great tits, with about 40 nests of each. Both species are short‐lived, hole‐nesting passerines, which defend a resource territory for breeding. The female builds the nest and incubates alone, but both parents feed the young.

### 2.2 Data collection

In spring 1997, we analyzed the bryophyte content of 34 great tit and 35 blue tit nests. We also analyzed bryophyte abundances in a total of 167 plots on the forest floor around nine of the trees with active nests (see below). Eight of the nests were included in the data set comparing blue tit and great tit nests in 1997, together with eight nests from the same nest boxes sampled in the subsequent year (1998). Sample number nine consisted of a great tit nest in a nest box analyzed for a pilot study in 1996 and a nest in the same nest box in 1998. We treated the 18 nests as independent samples. The tits were not ringed, but great tits are relatively short‐lived (Hõrak & Lebreton, 1998). Nest boxes were abundant in the study area and female great tits are known to move some distance between nest sites from 1 year to the next (Harvey et al., 1979).

We analyzed bryophytes in the nests when the nest lining was completed or when egg laying had started. Any egg(s) were laid aside and the nest was carefully removed from the box. The nests were then gently pried open in several places and from several angles, while care was taken not to destroy the nest, to visually estimate the proportion of each bryophyte species as a percentage of the total volume of bryophytes but later re‐calculated as a percentage of the whole nest. The nest and the eggs were then carefully returned to the nest box. There was no indication that this procedure caused any desertion.

**FIGURE 2:**
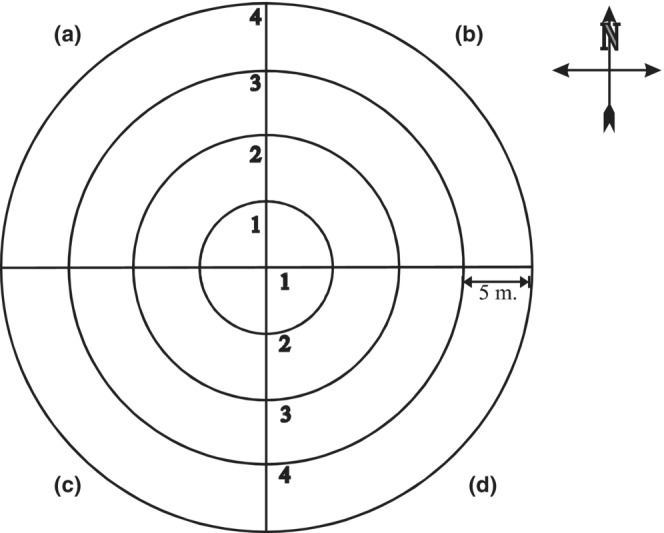
Schematic illustration of the vegetation analysis conducted around each nest box containing an active great tit nest. In each quadrant (a–d), there were five 2 m × 2 m^2^ plots, one randomly placed within each sector (1, 2, 3, 4), and one randomly placed in the whole quadrant. The radius of the circle was 20 m and the total area of the 20 plots was 80 m^2^, covering 6.4% of the total area of the circle (= 1256 m^2^).

Species abundances of bryophytes on the ground surrounding the nine nest boxes where great tits bred in both years were recorded as percentage cover in 2 × 2 m^2^ plots. We used restricted random sampling to place 20 plots around each of the nine nest boxes within a circular area with a radius of 20 m from the nest box. The plots therefore covered 6.4% of the circle area. The circle of 1256 m^2^ was divided into four quadrants, each with four sectors with a length of 5 m (Figure 2). In every quadrant, we placed one plot randomly in each sector and one plot randomly within the quadrant, giving five plots in each quadrant and 20 plots around each nest box. Thirteen of the plots were devoid of bryophytes, and the data set therefore consisted of 167 plots along with the 18 great tit nests. Assuming that bryophyte abundances were the same in both years, we only sampled the plots once.

### 2.3 Bryophyte nomenclature

The nomenclature of the bryophytes followed Frisvoll et al. (1995). Bryophytes were identified to species, except for *Brachythecium*, *Bryum*, *Dicranum*, *Hypnum*, *Pohlia*, *Plagiomnium*, *Plagiothecium*, *Polytrichum*, *Sphagnum,* and *Thuidium*, which were determined to genus. These are also referred to as species in the rest of the text. Liverworts were not included in the study as they were found in very small quantities in the pilot study in 1996.

### 2.4 Statistical analysis

We used R version 4.0.2. (R Development Core Team, 2020) for all statistical analyses. The statistical tests are two‐tailed unless otherwise specified, with an *α*‐level of 0.05.

To examine whether there were differences in bryophyte species composition between nests of blue tits (*n* = 35) and great tits (*n* = 34), we first extracted the gradient structure in the data sets by using two ordination methods in parallel, detrended correspondence analysis (DCA; Hill & Gauch Jr., 1980), and global nonmetric multidimensional scaling (GNMDS; Minchin, 1987), to confirm that structure axes were obtained (Økland, 1999). The DCA and GNMDS ordinations were relatively similar for the two first axes (Appendix S1). Therefore, we used the results of the DCA ordinations for all subsequent statistical analyses. The same ordination methods were used to examine whether bryophyte species composition differed between great tit nests (*n* = 18) and the epigeic bryophyte vegetation surrounding the nests (*n* = 167). The similarity of the DCA and GNMDS axes confirmed that the two first axes were structure axes (Appendix S1), and the results of the DCA ordination (distances between nests and plots along the two first axes) were therefore used in further analyses (see below).

We used the vegan package version 2.5–3 (Oksanen et al., 2019) for all ordination analyses. Prior to ordination, bryophyte species with a frequency below the median frequency were downweighted in proportion to their frequency (Eilertsen et al., 1990). We also weighted each matrix element with a power function (van Son & Halvorsen, 2014) to obtain a scale with a range, i.e., the ratio between the highest and lowest value, of 10:1 by using the weighting parameter w = 0.500 (Rydgren, 1993).

To examine whether blue tit and great tit nests differed in bryophyte species composition, we used GLM with identity link and Gaussian error to test whether the positions of the nests differed along the two first DCA ordination axes. We also tested whether the two tits differed in the number of bryophyte species they used in their nests using GLM with log link and Poisson errors. To test whether bryophyte species abundance differed between blue tit and great tit nests, we used GLM with identity link and Gaussian errors.

To test whether bryophyte species abundance differed between great tit nests and vegetation plots, we used GLMM with identity link and Gaussian errors (Bates et al., 2015). We included the nest box as a random factor to account for the spatial distribution of plots. Because bryophyte abundance was expressed as percentage cover, i.e., as strictly bounded but nonbinomial data, we logit transformed it (Warton & Hui, 2011) before the statistical analyses, and only species with a frequency higher than 3% in each data set were analyzed.

To examine whether great tits exhibited preferences or collected bryophyte species purely opportunistically, we used a randomization test. First, we calculated the observed mean M_0_ for all the DCA distances between the nests (*n* = 153). Next, 9999 random sub‐samples with 153 observations were drawn from the sample of all the local DCA distances between the tit nests and the respective plots (*n* = 334), and the mean M_1_ was calculated for each before we calculated the P‐value. In the randomization test, the P‐value for the test against one‐tailed alternative hypotheses was obtained by counting the number s of sub‐samples for which M_1_ < M_0_: *p* = .0001× (1 + s).

To examine whether great tits collected bryophytes near their nests, we conducted three tests based on distances between nests and plots along the first two axes in the DCA ordination. The first two of these were randomization tests. In the first test, we first calculated the observed mean M_0_ for the DCA distances between the nine nests built in the same nest boxes but in different years. Next, 9999 random sub‐samples with nine observations were drawn from the sample of all other DCA distances (*n* = 144) between the nests, and the mean M_1_ was calculated for each. In the second test, we first calculated the observed mean M_0_ for the 334 local DCA distances between nests and plots within the circles around the trees with nest boxes. Next, 9999 random sub‐samples with 334 observations were drawn from the sample of all the other DCA distances between nests and vegetation sample plots (*n* = 2672), and the mean M_1_ was calculated for each. In both randomization tests, the *p*‐value for the test against one‐tailed alternative hypotheses was obtained by counting the number s of sub‐samples for which M_1_ < M_0_: *p* = .0001× (1 + s).

In the third test, we used GLMM with identity link and Gaussian error to analyze the probable distances from the nest site at which great tits had collected bryophytes using the local DCA distances between nests and plots as the response variable, and the distance from the nest boxes (four levels, i.e., the different sectors), year (three levels) and their interaction as main factors. The nest box was included as a random factor to account for the spatial dependency of the plots. We started with the full model and pared it down using *p*‐values until we reached the minimal adequate model (Crawley, 2013).

## 3 RESULTS

### 3.1 Bryophyte species composition in the tit nests

**FIGURE 3:**
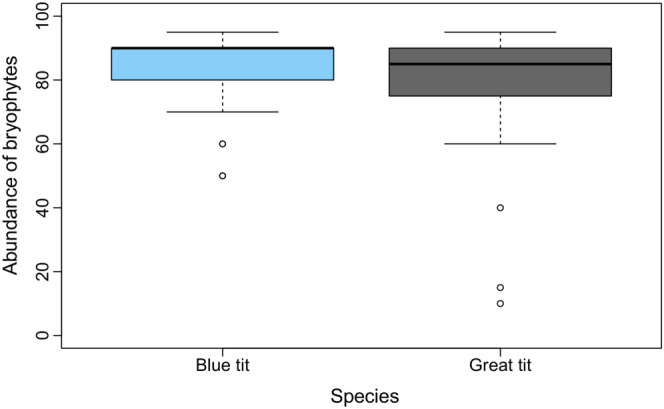
Boxplot of the abundance of bryophytes used in blue tit nests (*n* = 35) and great tits (*n* = 34). The horizontal line in each box is the median value, the box shows the interquartile range, and the dots are outliers beyond the whiskers.

**FIGURE 4:**
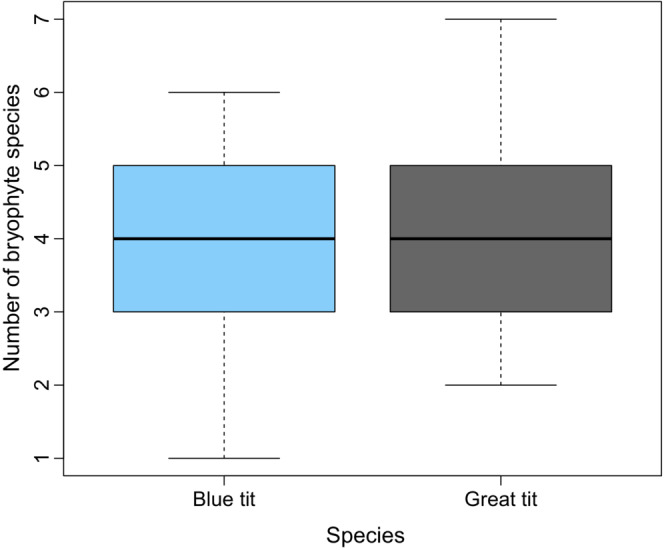
Boxplot of the number of bryophyte species used in blue tit nests (*n* = 35) and great tits (*n* = 34). The horizontal line in each box is the median value, the box shows the interquartile range, and the whiskers show the range of the data.

Both blue and great tits used bryophyte material abundantly when building their nests: bryophytes made up 90% of blue tit nests and 85% of great tit nests (median values, see Figure 3). Bryophytes were always used together with nonbryophyte material, such as feathers and grasses for blue tits and hair for great tits. The two species used the same number of bryophyte species (*p* = .91), with a median of four (Figure 4).

**FIGURE 5:**
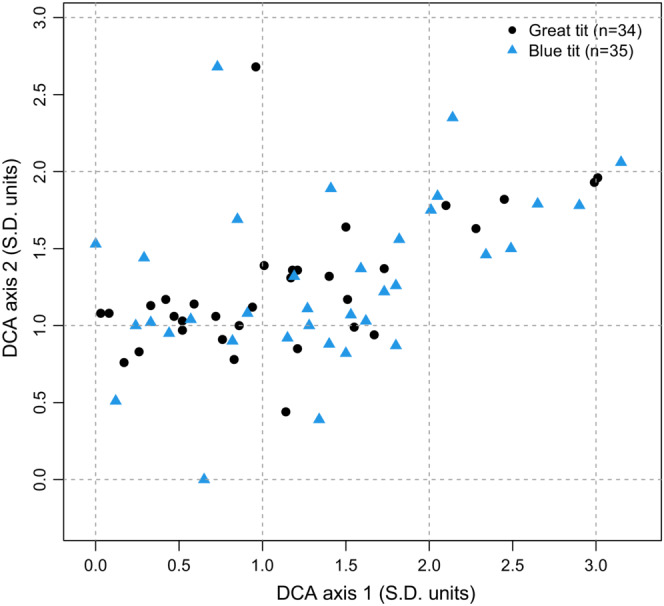
DCA ordination of the bryophyte species composition of blue and great tit nests.

The bryophyte species composition of nests built by blue tits and great tits was fairly similar (Figure 5). The positions of the two species' nests along the two first DCA ordination axes did not differ significantly (DCA axis 1, *p* = .23; DCA axis 2, *p* = .73). The bryophytes most frequently used by both bird species were the pleurocarpous taxa *Brachythecium* spp., *Hylocomium splendens*, *Pleurozium schreberi*, *Rhytidiadelphus squarrosus*, *Sanionia uncinata, and the acrocarpous Dicranum* spp. (Table 1). Both tits used a total of 15 species, but about one‐third of these were used so rarely, or in such small amounts, that this can probably be regarded as incidental usage (Table 1).

**TABLE 1:** Occurrence of bryophyte species in nests of blue tits and great tits built in 1997.

		Great tit			Blue tit		
	Growth form	Freq.	Mean	Range	Freq.	Mean	Range
*Brachythecium spp*.	*P*	44	4	0–18	54	24	0–85
*Cirriphyllum piliferum*	*P*	12	6	0–15	17	20	0–60
*Climacium dendroides*	*P*	3	1	0–1	–	–	–
*Dicranum spp*.	*A*	24	2	0–11	43	5	0–36
*Hylocomium umbratum*	*P*	9	9	0–20	3	20	0–20
*Hylocomium splendens*	*P*	47	10	0–45	37	26	0–81
*Hypnum spp*.	*P*	3	1	0–1	6	2	0–2
*Leucodon sciuroides*	*P*	–	–	–	3	55	0–55
*Plagiomnium spp*.	*P*	–	–	–	9	1	0–1
*Plagiothecium spp*.	*P*	6	3	0–5	20	1	0–2
*Pleurozium schreberi*	*P*	82	24	0–80	66	28	0–83
*Polytrichum spp*.	*A*	15	2	0–4	3	1	0–1
*Ptilium crista‐castrensis*	*P*	15	17	0–80	17	21	0–87
*Rhytidiadelphus loreus*	*P*	6	26	0–50	6	28	0–55
*Rhytidiadelphus squarrosus*	*P*	76	45	0–89	57	32	0–85
*Sanionia uncinata*	*P*	41	24	0–94	40	24	0–94
*Sphagnum spp*.	*S*	3	1	0–1	–	–	–

*Note*: Growth form—A, Acrocarpous, P, Pleurocarpous; S, Spagnum; Freq.—percentage of nests where the bryophyte species occurred; *n* = 34 for the great tit and *n* = 35 for the blue tit; Mean—arithmetic mean of the abundance of each bryophyte species, calculated from the nests in which the species was present; Range—range of values.

### 3.2 Bryophytes in great tit nests and on the ground in the surrounding areas

**FIGURE 6:**
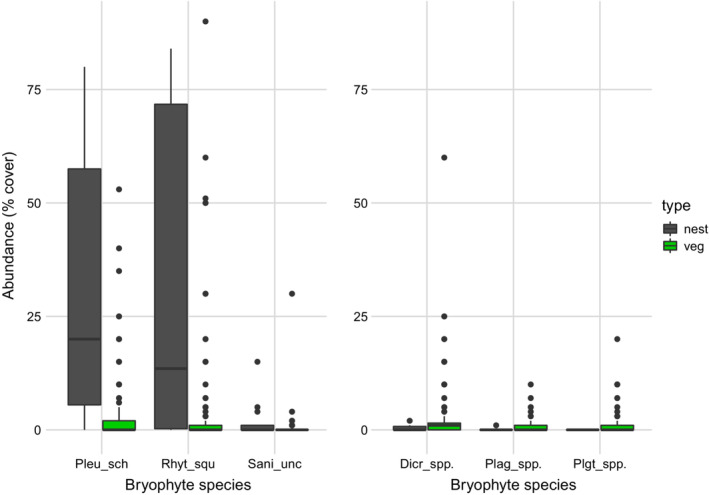
Six bryophyte species showing significantly higher (left) or lower (right) abundance (cover) in great tit nests (*n* = 18) than in the vegetation (veg) in surrounding areas. Higher: Pleu sch = *Pleurozium schreberi*; Rhyt_squ = *Rhytidiadelphus squarrosus*; Sani_unc = *Sanionia uncinate*. Lower: Dicr_spp = *Dicranum* spp.; Plag_spp = *Plagiomnium* spp.; Plgt_spp = *Plagiothecium* spp.

We studied the abundance of bryophyte species in 18 great tit nests and in a total of 167 vegetation plots in surrounding areas. A total of 26 bryophyte species were found, 16 in the tit nests and 24 species in the plots. Two bryophyte species were recorded in tit nests only (in one nest each). Ten species were found in the plots only. Four of these were common and occurred in more than 7% of the plots. The median number of bryophyte species was four in great tit nests and five in the plots. Of the 17 species found in more than 3% of the total number of samples, three species showed significantly higher abundance in the tit nests than in the plots, i.e., *Pleurozium schreberi*, *Rhytidiadelphus squarrosus*, and *Sanionia uncinata*, and three species showed significantly lower abundance, i.e., *Dicranum* spp., *Plagiomnium* spp., and *Plagiothecium* spp. (Figure 6). For 11 species, there was no significant difference in abundance between nests and plots.

The choice of bryophytes by great tits was not purely opportunistic, but showed a preference for certain bryophyte species, as revealed by the significantly shorter DCA distances between the nests than between the nests and the respective local plots (m_0_ = 1.08, *n* = 153, vs. m_1_ = 1.48, *n* = 334; *p* < .001). This means that there was far more similarity between the nests than between the nests and the epigeic bryophyte community in the surroundings.

**FIGURE 7:**
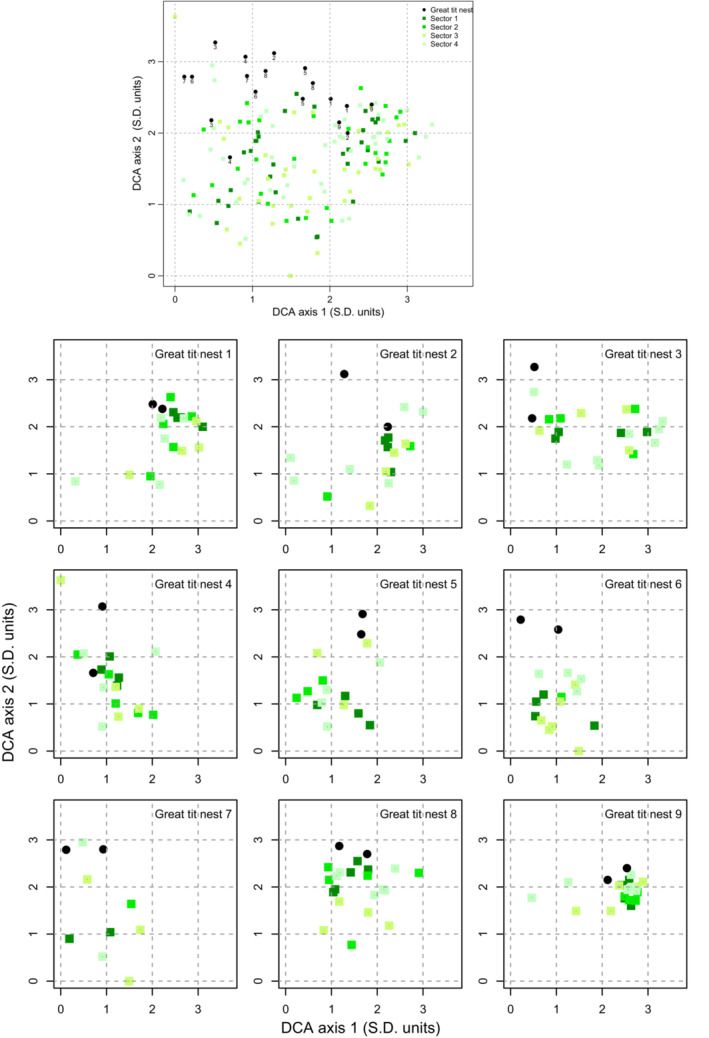
DCA ordination of the bryophyte species composition of 18 great tit nests and the 167 plots in surrounding areas. The plots were in four different sectors around each nest, positioned at increasing distances from the nests. In the upper panel, the numbers next to the nest positions show the nest's identity (box number). In nine cases, the same nest box was sampled in two different years. The lower panels show the results of the same DCA ordination for each of the nine nest boxes and plots. Each panel shows values for plots in all four sectors for the two nests in the same nest box. In some cases, plots are hidden because they have the same value as other plots.

Great tits seemed primarily to use bryophytes found close to the nest (Figure 7). First, the DCA distance was almost significantly shorter for nests built in the same box but in different years than for nests from different boxes (m_0_ = 0.82, *n* = 9, vs. m_1_ = 1.09, *n* = 144; *p* = .067). Second, the bryophyte species composition of the nests was significantly more similar to that of the respective local plots than to that of more distant plots (plots surrounding the other nests), as revealed by the much shorter DCA distances (m_0_ = 1.48, *n* = 334, vs. m_1_ = 1.67, *n* = 2672; *p* < .001). Third, the bryophyte species composition of plots in the sector nearest to a nest box (radius of 5 m from the tree with the box) was significantly more similar to the bryophyte species composition of the nests (shorter DCA distances) than the species composition of the more distant sectors (*p* = .002).

## 4 DISCUSSION

Our main findings were that the use of bryophytes as nest materials was quite similar in blue tits and great tits, and that great tits collected bryophyte species selectively and not at random in proportion to availability within their territory. Finally, great tits probably collected most of the bryophytes within a distance of only 5 m from the nest.

### 4.1 Blue tits and great tits used similar species of bryophytes

Blue tit and great tit nests contained about the same number of bryophyte species, generally the same species and in similar abundances. Pleurocarpous mosses dominated. These have highly branched, interwoven stems (Shaw et al., 2003). This seems to be a common pattern in nest building by the two tit species (Wesołowski & Wierzcholska, 2018). The use of a few dominant bryophyte species in the nest is also common in other birds that use large quantities of bryophytes such as the prothonotary warbler, *Protonotaria citrea* (Blem & Blem, 1994), pied flycatcher*, Ficedula hypoleuca* (Briggs & Deeming, 2016), and many other species (Glime, 2017b).

In general, blue tits forage higher above the ground than great tits, which spend much time on the ground (Slagsvold & Wiebe, 2007). This seems to be reflected in their use of nest materials; blue tits use more epiphytic bryophytes collected from tree trunks, and great tits mainly use epigeic species (Glądalski et al., 2021; Henze, 1962; Wesołowski & Wierzcholska, 2018). Thus, the nest materials used by the two species will probably reflect the comparative availability of epiphytic and epigeic bryophytes in their territories. This may explain why there were noticeable differences between the two tit species in Białowieża National Park in Poland (Wesołowski & Wierzcholska, 2018). Our results, with only small differences between the two tits, are similar to those from urban parks and deciduous forest in Łódź, Central Poland (Glądalski et al. (2021). The results may be typical of habitats with poor bryophyte epiphytic flora such as our study site in Norway, and probably many other boreal areas.

### 4.2 Selective choice of bryophytes by great tits

Our results show that the great tits are selective in their use of bryophytes as nest materials. First, there was closer similarity in bryophyte species composition across all the great tit nests than in the epigeic bryophyte species composition across the sample plots surrounding the tit nests. This was largely because the great tits showed a disproportionally strong preference for the pleurocarpous mosses *Pleurozium schreberi*, *Rhytidiadelphus squarrosus*, and *Sanionia uncinata*, and a disproportionally weak preference for the acrocarpous *Dicranum*, and pleurocarpous *Plagiomnium*, and *Plagiothecium* species. Our results add to recent research from Poland showing that great tits (and blue tits) are skilled “bryologists” with strong preferences for certain species (Glądalski et al., 2021; Wesołowski & Wierzcholska, 2018).

Usually, blue tits and great tits build the nest cup on a thick foundation of bryophytes. This supports the nest, and may also insulate its contents and absorb water, avoiding moisture in the nest cup (Biddle et al., 2019; Deeming et al., 2020; Wesołowski & Wierzcholska, 2018). However, it is not evident what makes certain species of bryophytes more suitable than others. There are several possible hypotheses (cf. Deeming & Mainwaring, 2015; Glime, 2017b). One hypothesis assumes that variation in the microhabitats in which the bryophytes are found affects how difficult they are to reach (Glądalski et al., 2021). Some species may grow in more exposed sites than others, possibly affecting the risk of predation. During nest building, females may be particularly vulnerable to predation by avian raptors, as has been shown for pied flycatchers *Ficedula hypoleuca* in our study area (Slagsvold & Dale, 1996).

A second hypothesis assumes that the time it takes to collect different bryophytes varies, for instance depending on how difficult it is to pick up bundles of suitable sizes (Wesołowski & Wierzcholska, 2018). Longer handling times may increase the risk of predation by avian raptors, particularly when collecting items on the ground (Slagsvold & Dale, 1996). This may account for differences between the tit species in bryophyte use (Wesołowski & Wierzcholska, 2018), assuming that the blue tit avoids spending time on the ground where it has less foraging experience than the great tit. However, constraints related to predation risk can hardly be a general explanation because epigeic bryophytes were not chosen at random, at least not by great tits.

A third hypothesis is that great tits prefer bryophyte species with a higher water‐absorbing capacity. Nest materials differ in their absorbing capacity, and nests consisting of large quantities of bryophytes generally absorb much water and dry out slowly (Biddle et al., 2019; Slagsvold, 1989). However, the bryophyte species differ in their water storage capacities and evaporation rates (Busby et al., 1978; Elumeeva et al., 2011; Michel et al., 2012; Proctor et al., 2007), but so far, there is no indication that our studied tit species select bryophytes based on differences in water‐absorbing capacity between the bryophytes (Wesołowski & Wierzcholska, 2018). A fourth hypothesis is that the different structural properties of bryophyte species result in varying insulation properties (e.g., Deeming & Mainwaring, 2015). Bryophytes are important as insulators in birds' nests (Deeming et al., 2020), but their suitability may differ, but to our knowledge, this has so far not been examined. Tits generally line the nest cup with a thick layer of materials with excellent insulation properties (fur, hair, and feathers), but the bryophyte layer under the nest cup and in the nest wall may be important, for instance for maintaining air gaps (Glądalski et al., 2021).

A fifth hypothesis is that preferences are related to the suitability of bryophytes as structural materials, both to construct a nest with the desired form and to avoid early collapse of the nest cup as the nestlings become older and more active (Wesołowski & Wierzcholska, 2018). Pleurocarpous mosses with their highly branched and interwoven stems are probably better building materials than acrocarpous mosses, which show little or no branching. A sixth hypothesis is that preferences may be related to potential food sources, as shown for the Japanese tit (*Parus minor*), which has higher fledging success with bryophyte nest material containing moths (Glime, 2017a; Hamao et al., 2016). A seventh hypothesis is that tits avoid unbranched bryophytes because ectoparasites like hen fleas, reducing the birds´ breeding success (Heeb et al., 2000), may hide more readily in such substrates.

Finally, the eighth hypothesis (e.g., Clark & Mason, 1988; Wimberger, 1984) is that birds use green plants in their nests that contain secondary compounds that deter avian ectoparasites. Originally, this nest protection hypothesis concerned vascular plants that were not part of the nest structure properly (Wimberger, 1984). However, birds' preferences for specific bryophyte species may be related to their production of secondary metabolites with antimicrobial, antifungal, or antibacterial bioactivity (Horn et al., 2021; Klavina et al., 2015), which may reduce populations of pathogens and ectoparasites in the nest environment. Little is known about how the presence of different bryophytes in a nest, influences living conditions for pathogens and parasites. A recent study of a generalist hummingbird species by Fontúrbel et al. (2020) does, however, support the hypothesis. In our study, two of the bryophytes strongly preferred by great tits, *Pleurozium schreberi* and *Rhytidiadelphus squarrosus*, have shown antimicrobial, antifungal, or cytotoxic effects (Nikolajeva et al., 2012; Veljić et al., 2008; Wolski et al., 2021). However, more research is still needed on the relationships between bryophytes and the pathogens and parasites in bird nests (Glime, 2017b).

### 4.3 Great tits collected bryophytes close to their nests

First, we found great similarity between bryophyte species composition in great tit nests in the same nest box, and thus within the same local microhabitat, across years. This indicates that great tits fly short distances when collecting nest material and as predicted, given the assumption that the abundance of different bryophyte species within a local forest area is rather similar in years without disturbances (Rydgren et al., 2004). Next, we compared the bryophytes found in great tit nests with the epigeic bryophytes growing in the plots located within 20 m of each tit nest. These results indicated that most of the materials were collected within close range and probably within only 5 m. Thus, the tits seemed to minimize the time and energy spent collecting by flying as short a distance as possible but at the same time seeking specific bryophytes. Our results are consistent with the general view that nest building in birds is costly (Mainwaring & Hartley, 2013), which was supported by supplementary feeding experiments in the two tit species (Mainwaring & Hartley, 2009; Smith et al., 2013).

### 4.4 Heritability and learning of preferences

Increasing evidence suggests that birds' nest material preferences are not entirely genetically predetermined, since they can adjust nest construction based on experience (Breen, 2021; Breen et al., 2016; Camacho‐Alpízar et al., 2021). To our knowledge, to what extent preferences for certain bryophyte species are inherited is not known. However, some information exists on the use of feathers as lining materials. Cross‐fostering between great tits and blue tits in the field showed that the use of feathers is not a result of cultural transmission (Aasen & Slagsvold, 2020). In another study of blue tits, repeatability in the use of feathers by individual females across years was low (Järvinen et al., 2017), as was the similarity between mother and daughter, both in feather use and in nest depth (Järvinen et al., 2017; O'Neill et al., 2018).

## 5 CONCLUSIONS AND RECOMMENDATIONS

In the present study, we demonstrated the importance of obtaining quantitative data on the specific materials found in bird nests, as emphasized by many authors (e.g., Biddle et al., 2017; Deeming & Mainwaring, 2015). In addition, we compared the bryophyte species found in tit nests with the bryophyte species composition in the immediate surroundings of the nests. The study design made it possible to reject the null hypothesis that bryophyte choices by great tits were random.

We advocate closer collaboration between ornithologists and bryologists to investigate the bryophyte species composition of bird nests and not just the total mass or volume of bryophytes. We also advocate adopting a sampling design like ours to obtain multivariate data sets that can be analyzed by ordination. As with other data analyses, there may sometimes be a mismatch between the model and the data, and ordination may produce spurious axes (Økland, 1990). To ensure that the ordination axes represent the true structure, two ordination methods from different families should be used in parallel to enhance the detection of artifacts in the results (van Son & Halvorsen, 2014).

More insights can be gained by conducting choice experiments (Briggs & Mainwaring, 2019; McGowan et al., 2004; Surgey et al., 2012). Alternatively, the moss layer in some tit nests could be exchanged for a similar layer of nonpreferred bryophyte species to study whether the bryophyte content affects the insulation properties of the nest, the risk of collapse of the nest structure, the abundance of fleas, and overall breeding success. Heritability may be studied in the same way as has been done for feathers, by cross‐fostering between and within species, by comparing nest building between mothers and daughters, and by comparing nest building by individual females both within and between breeding seasons.

## AUTHOR CONTRIBUTIONS

**Knut Rydgren:** Conceptualization (equal); formal analysis (lead); investigation (supporting); methodology (equal); project administration (equal); writing – original draft (lead). **Bendik Indreeide:** Investigation (lead); writing – original draft (supporting). **Tore Slagsvold:** Writing – original draft (supporting). **Helene Lampe:** Conceptualization (equal); investigation (supporting); project administration (equal); writing – original draft (supporting).

## CONFLICT OF INTEREST STATEMENT

The authors declare that they have no conflict of interest.

## Supporting information


Appendix S1.

## Data Availability

Data Accessibility: Data available via the Dryad Digital Repository DOI https://doi.org/10.5061/dryad.tqjq2bw3v
