# Supplementary material for: Nest building in titmice Paridae: Selectivity in bryophyte use
Source: Ecol Evol. 2023 Mar 8;13(3):e9852. doi: 10.1002/ece3.9852 (PMC9994477; doi:10.1002/ece3.9852)
Supplement: Supplementary file 1 — Appendix S1. [file ECE3-13-e9852-s001.docx]

**Supplementary material**

**Appendix S1.** Ordination analyses.

The gradient structure of the two data sets (34 great tit and 35 blue tit nests, and 18 great tit nests and 167 plots on the ground) was extracted by parallel use of detrended correspondence analysis (DCA; Hill & Gauch, 1980) and global non-metric multidimensional scaling (GNMDS; Minchin, 1987) as implemented in the vegan package version 2.5-3 (Oksanen et al., 2019). DCA was run with 4 rescaling cycles. GNMDS was run using the function monoMDS in the vegan package, with the Bray-Curtis dissimilarity index. The maximum number of iterations was 500, and convergence tolerance criterion was set to 10^–7^. GNMDS solutions were obtained from 2000 different random starting configurations, of which more than two had to give identical minimum stress solutions. This best solution was subjected to varimax rotation using principal component analysis (PCA) and the resulting axes were re-scaled to half-change (H.C.) units. We solved for two-, three- and four-dimensional GNMDS solutions.

For each of the two data sets, we compared pairs of axes using Spearman’s rank correlation coefficient r_s_. For the data set of 34 great tit and 35 blue tit nests, the first axis of DCA was strongly correlated with the first axis of all the three GNMDS solutions (0.94 ≤ |r_s_| ≤ 0.98; all with P < 0.001). The correlation was weaker for the second DCA axis and the second axis of the three GNMDS solutions (0.18 ≤ |r_s_| ≤ 0. 37), and only significant for the two-dimensional solution (P=0.002). The second DCA axis was in fact more strongly correlated with the third GNMDS axis of the three-dimensional solution (r_s_ = 0.72) and with the fourth GNMDS axis of the four-dimensional solution (r_s_ = 0.58).

For the data set of 18 great tit nests and 167 plots on the ground, the first axis of DCA and the first axis of all three GNMDS solutions were strongly correlated (0.61 ≤ |r_s_| ≤ 0.65; all with P < 0.001) but the first DCA axis was even more strongly correlated with the second GNMDS axes for all the GNMDS solutions (0.67 ≤ |r_s_| ≤ 0.72). The second DCA axis was strongly correlated with the second axis of all three GNMDS solutions (0.35 ≤ |r_s_| ≤ 0.40; all with P < 0.001) but the second DCA axis was even more strongly correlated with the first GNMDS axis for all the GNMDS solutions (0.78 ≤ |r_s_| ≤ 0.80). The first and second DCA axes are shifted compared to the GNMDS solutions, but both axes of all ordinations appear to represent the true gradient structure. Since we used the Euclidean distance between plots and nests, the axis shift had no influence on the outcome.

**References**

Hill, M. O., & Gauch, H. G., Jr. (1980). Detrended correspondence analysis: an improved ordination technique. *Vegetatio, 42*, 47-58.

Minchin, P. R. (1987). An evaluation of the relative robustness of techniques for ecological ordination. *Vegetatio, 69*, 89-107.

Oksanen, J., Blanchet, F. G., Friendly, M., Kindt, R., Legendre, P., McGlinn, D., . . . Wagner, H. (2019). *vegan: Community Ecology Package Version 2.5-3*. <http://cran.r-project.org>: The R foundation for statistical computing.
